# Construction of consumer satisfaction evaluation index system for green products based on online comments

**DOI:** 10.1371/journal.pone.0322470

**Published:** 2025-04-29

**Authors:** Changlu Zhang, Zihao WEI, Jian Zhang, Liqian Tang

**Affiliations:** 1 School of Economic & Management, Beijing Information Science & Technology University, Beijing, China; 2 School of Computer Science, Beijing Information Science & Technology University, Beijing, China; 3 Beijing Key Lab of Green Development Decision Based on Big Data, Beijing, China; Zhejiang Gongshang University, CHINA

## Abstract

The promotion of green product consumption and its transformation towards low-carbon alternatives is essential for implementing new developmental paradigms and achieving carbon neutrality objectives. This study employs text mining techniques to analyze user online comments from e-commerce platforms, focusing on consumer satisfaction regarding green products. Utilizing the KeyBert model, relevant keywords were extracted from user feedback, followed by the training of keyword vectors using Word2Vec. K-means clustering was then employed to develop a comprehensive system of consumer satisfaction evaluation index system for energy-saving air conditioning products on the JD platform. The findings reveal that consumers prioritize functionality, service quality, aesthetic appeal, pricing, logistics, and installation in their evaluations. It is recommended that manufacturers enhance installation procedures, refine aesthetic designs, and emphasize functional advantages to elevate consumer satisfaction. However, this study is limited by its focus on a singular product category and necessitates further research incorporating a broader dataset to validate these findings. Future investigations should consider a wider range of green products and leverage diverse data sources to enrich the analysis.

## 1. Introduction

As environmental issues become increasingly critical, green consumption has garnered significant public interest. Driven by the concept of sustainable development, substantial progress has been achieved in promoting green consumption. During China’s “13th Five-Year Plan” period, the overall green consumption level among urban and rural residents exceeded 25% [1–2]. The rapid economic growth and rising living standards have heightened public awareness and interest in green products. To stimulate the adoption of these products, various countries and regions have instituted unified green product certifications through authoritative bodies, aiming to enhance consumer willingness to engage in green consumption behaviors [3–4].

Despite these advancements, research on the green consumption experiences and satisfaction levels of residents is limited, yet it is crucial for supporting supply-side structural reforms and facilitating high-quality development. The rapid rise of e-commerce has introduced multi-dimensional dynamics in online shopping, culminating in a new consumption paradigm [5]. The increasing variety of green products available on e-commerce platforms has further fueled consumer enthusiasm for expressing their experiences and emotions online [6].

Online reviews represent active consumer feedback post-purchase, offering rich and valuable insights into consumer sentiments. These reviews are characterized by substantial data volume, diverse information, and easy accessibility. They not only inform prospective buyers but also provide vital data for the enhancement of green products [7–8]. Merchants can utilize these insights to better understand consumer needs and satisfaction levels, ultimately improving product offerings and refining marketing strategies. Consequently, in the digital intelligence era, online reviews have emerged as a pivotal foundation for businesses to innovate and for consumers to make informed purchasing decisions [9].

However, existing research on product satisfaction predominantly relies on questionnaire surveys and traditional satisfaction models. The exploration of consumer satisfaction through the analysis of online reviews for green products remains scarce. Traditional models typically impose a predefined satisfaction index system through surveys, which can introduce uncertainty due to factors such as the validity of the indicators, survey scale, costs, and feedback timeframes [10–12]. In contrast, the subjective feelings reflected in actively shared online review texts provide a more accurate representation of consumer sentiments. Therefore, evaluating consumer satisfaction regarding green products through text mining methods holds significant potential for fostering green consumption and achieving high-quality development.

This article aims to analyze online review texts of green products on leading e-commerce platforms using text mining techniques to construct a consumer satisfaction evaluation index system, thereby enhancing the scientific rigor and objectivity of the evaluation process.The structure of this article is as follows: The literature review reviews and synthesizes existing research on consumer satisfaction and text mining. The model construction develops a green product consumer satisfaction evaluation model based on text mining. The empirical research presents empirical research findings.

## 2. Literature review

Based on online comment texts, using text mining methods to conduct research on consumer satisfaction evaluation of green products can objectively obtain the dimensions that consumers are concerned about and their real consumption experience. This is the key to promoting green consumption and promoting green development. Therefore, we have reviewed existing literature from two perspectives: consumer satisfaction and online comment text mining.

Consumer satisfaction refers to the degree to which consumers are satisfied with a certain product or service [13].Consumer satisfaction is a person’s feelings after consuming a product or service and is compared to their expectations. Consumer satisfaction can be influenced by Service Quality, Product Quality and Purchasing Decisions. Domestic and foreign scholars have conducted extensive research on consumer satisfaction. According to the different methods and models of data acquisition, it can be roughly divided into three aspects:

The first is to construct a consumer satisfaction index model using traditional methods such as interviews and questionnaires. Representative achievements such as Wang Hongxin and Liu Yuhui obtaining consumer online fresh agricultural product data through survey questionnaires. They proposed seven factors and corresponding hypotheses that affect consumer satisfaction based on the Chinese Consumer Satisfaction Index model, and then conducted statistical analysis [14]. (2015) Li Ning et al. (2019) obtained basic data by distributing survey questionnaires to consumers and constructed a satisfaction factor model for consumers purchasing fresh agricultural products online [15]. Li Wen et al. (2020) distributed questionnaires to fresh agricultural product consumers under the O2O model and studied the relationship between factors affecting consumer shopping satisfaction through correlation analysis [16]. Yang Hongyan et al. (2020) conducted mining and analysis on three-year consumer food safety satisfaction survey data based on association rules, and determined the influencing factors of consumer satisfaction with food safety [17]. Lee et al. (2019) conducted a study through distributing survey questionnaires and found that the webpage design of e-commerce platforms has a positive impact on consumer satisfaction, which can enhance consumer purchase intention by improving the design level [18–19]. Birjoveanu (2019) first qualitatively analyzed the willingness and behavior of e-commerce consumers in Liaoning Province, and constructed a theoretical model. Then, through quantitative analysis of the questionnaire survey data, he used model fitting and hypothesis testing methods to verify that various factors such as the design elements of the e-commerce platform interface and the security of network information have a significant impact on consumer willingness and satisfaction [20].

The second is to use the information in online comment text data to construct a traditional consumer satisfaction index model. Representative achievements such as Wei Helin et al. (2020) focused on five aspects of product characteristics and consumer reviews, and used stepwise regression method to explore the influencing factors of online word-of-mouth on team tourism product online booking. At the same time, they explored the relationship between the dependent variable and indicator characteristics [21]. Zhang Yanfeng et al. (2019) constructed a technology acceptance model and a linear regression model to study the factors affecting comment time. The research results found that the model can effectively discover the relationship between consumer online comment behavior and comment time, which helps to discover and predict the characteristic patterns of consumer comment time [22]. Geebren et al. (2021) demonstrated a significant positive impact of trust on customer satisfaction using a partial least squares structural equation model based on 659 satisfaction data from electronic banking. And the relationship between trust and intermediary structure assurance, service quality, and customer satisfaction [23].

The third is to use online comment text information to construct a consumer satisfaction evaluation model by calculating emotional tendencies. Representative achievements such as Sun Baosheng et al. (2022), based on online tourism review data and online text mining technology, constructed a tourist satisfaction evaluation index system and evaluation model, and quantitatively evaluated tourists’ ecotourism satisfaction based on a tourism sentiment lexicon [24]. Geng Xiaoli et al. (2019) constructed an LDA topic model through user online comments and conducted sentiment analysis, based on which they explored the most important factors affecting online user purchase satisfaction [25]. Zhang Zhengang et al. (2022) extracted product attributes from online comments and conducted sentiment analysis. Based on this, they constructed a classification model to identify different attribute requirements [26]. Zheng Songyin et al. (2022) constructed a digital service vocabulary by collecting comments from museum users and extracted aspect level statements. Then they conducted sentiment classification on aspect level statements, and finally analyzed the factors influencing user experience based on the classification results [27]. Zhao Yuqing et al. (2020) improved the traditional KANO model and combined it with online comments to achieve objective measurement of user demand acquisition and demand fulfillment [28]. Tu Min (2020) used the method of online comment clustering to calculate product satisfaction and constructed a satisfaction quartet, providing a new research approach for measuring user satisfaction [29]. Hao et al. (2021) analyzed the logistics factors that affect consumer satisfaction with JD agricultural products through text mining technology and optimized the e-commerce delivery path [30].

Through reviewing relevant literature both domestically and internationally, it has been found that researchers use different methods to study consumer satisfaction in different fields. At present, domestic and foreign scholars have gradually shifted their research methods on consumer satisfaction from traditional methods such as questionnaires and interviews to online comment text mining methods. At the same time, scholars have paid extensive attention to consumer purchasing behavior and preferences of products with certification marks. However, the research mainly focuses on the pre-purchase behavioral intentions of consumers, and there is still a lack of relevant research on post purchase satisfaction of labeled products.

Online comment content on e-commerce platforms is currently a research hotspot in the field of management decision-making and an important direction of information mining. In recent years, the scope of research on online comments has been continuously expanding, and research results have emerged. According to the different research content, it can be roughly divided into four aspects: number of comments, quality of comments, length of comments, and sentiment of comments [31].

In terms of the quantity characteristics of online comments: Cui et al. found that the number and validity of online comments have a significant impact on the sales of newly launched products when exploring the impact of online comment data on the sales of newly launched products [32].Shi Wenhua, Zhong Biyuan, and Zhang Qi (2017) investigated the relationship between movie box office revenue and the number of online film reviews. Their comparative analysis of online film reviews and online short reviews revealed that the quantity of online short reviews significantly influences box office performance [33].

Yang Xian et al. demonstrated the relationship between the number of online comments and consumer purchase intention by constructing a model of the relationship between the number of comments and purchase intention [34]. Niu Gengfeng et al. studied the impact mechanism of comment quality and quantity on consumer purchase intention through experimental design. Their research findings show that both the quantity and quality of comments affect consumer purchasing decisions, and have varying degrees of impact on individuals with different cognitive needs [35].

In terms of the quality characteristics of online comments: Sun Jin et al. found that the higher the quality of comments, the more it can affect consumers’ online purchasing decisions [36]. Cao Yu and others conducted research based on the theory of regulatory orientation, which showed that consumer cognition, consumer purchase intention, and comment quality are closely related. They also found that high-quality online reviews have a greater impact on consumer purchasing decisions [37]. Hong Fei et al. studied college student consumers and proposed a theoretical model of the impact of online comments on college student online shopping consumption. Research has shown that the higher the quality of online comments, the more useful value consumers gain, and the more it can promote consumer purchase intention [38]. Huo Hong et al. studied the importance of perceived risk in the quality of online comments and demonstrated through empirical research that high-quality comments can help consumers avoid perceived risk to a certain extent [39].

In terms of the length characteristics of online comments, Chevalier et al. found that the length of online comment text positively affects the usefulness of comments [40]. Li Ang et al. found through research that the more words online comments have, the higher their usefulness [41]. Ye et al. introduced online review research into the hotel industry and found that the quantity and quality of online review texts have a certain impact on hotel sales by analyzing relevant online review texts [42].

In terms of emotional characteristics of online comments, Zhao Tianrui et al. utilized deep learning technology to construct a Korean film review sentiment dictionary, and then formed the sentiment analysis model, which effectively completed sentiment analysis of Korean short texts [43]. Wang Yang conducted online comment data mining, emotion analysis and opinion extraction for spray products in small and medium-sized agricultural equipment. On the basis of fully obtaining user attribute opinions, he proposed corresponding improvement strategies [44]. Munuswamy et al. extracted valuable information from social user comments using sentiment rating prediction methods. They calculated the sentiment value of a single user's product based on a sentiment dictionary, thereby predicting project ratings and calculating the reputation of the product [45]. Wang Weina combines subjective online text and sentiment analysis techniques with consumer satisfaction knowledge. She focused on the important attributes of the relevant products, which provide reference value for the subsequent optimization of the products [46]. Liu Yulin et al. established an emotional index based on emotional tendencies and dynamically monitored emotional changes in online comment texts to grasp the emotional trends of e-commerce platforms [47].

In light of these insights, this study not only addresses the gap in the existing literature regarding post-purchase satisfaction of green products but also offers a novel perspective by emphasizing the significance of extracting authentic consumer experiences through online comment text mining. Unlike prior research predominantly focused on pre-purchase intentions, this investigation delves into the specific determinants of consumer satisfaction within the realm of green product consumption. The insights gained from this research can significantly inform businesses on strategies to optimize their offerings in the green market, thus fostering sustainable consumption patterns. Furthermore, the findings of this study pave the way for future research endeavors aimed at exploring the multifaceted dimensions of consumer satisfaction in environmentally friendly product categories.

Thus, online comment text mining provides a robust method for understanding consumer satisfaction and can offer actionable insights for green product optimization. Sentiment analysis, in particular, serves as a powerful tool for evaluating satisfaction levels and identifying improvement areas, ultimately contributing to consumption upgrading and high-quality development. The emphasis on post-purchase satisfaction in the context of green products is underscored as a distinct contribution to the literature. In contrast to prior research, which predominantly explores general drivers of consumer satisfaction or is focused on pre-purchase intentions, this work investigates specific determinants of satisfaction within green product consumption. By providing an authentic consumer perspective, this approach aligns with broader goals of fostering sustainable consumption patterns and advancing high-quality development within green markets.

## 3. Model construction

We crawled the user review data of green products on e-commerce platforms and used natural language processing technology to denoise and segment the review text. Using the KeyBert algorithm to extract keywords from comment texts. Then the Word2Vec tool was used to train keyword word vectors. Finally, the K-means algorithm was used for keyword clustering and ultimately to construct a green product consumer satisfaction evaluation index system.

### 3.1. Keyword extraction for online comments based on KeyBert

KeyBERT is a keyword extraction method developed through research and development led by Mararten Grootendors. The model enables users to extract keywords or key phrases from the given text and embed sentences or documents into highdimensional vector representations using BERT [48].The basic steps of the Key Bert method are as follows:

Firstly, encode the comment text. We record the comment text that requires keyword extraction as t. And use a pre-trained Bert model to encode the text, which can obtain vector representations of each word in the text. These vectors form a matrix, as shown in formula (1).

- **Input**: The comment text t.
- **Output**: A matrix of word vectors H, where each vector *h*ᵢ represents the i-th word in the comment text.

$$H=[h_{1},h_{2},...,h_{n}]$$ (1)

The *h*ᵢ in the formula represents the vector of the i-th word in a comment text.

Secondly, the vectorization of the comment text. In order to obtain the vector representation *V(t)* of the comment text, it is necessary to summarize the vectors of each word. A common method is to perform an average operation on word vectors, which involves adding up all word vectors and dividing them by the number of words n. The specific calculation is shown in formula (2).

- **Input**: The matrix of word vectors H and the number of words n.
- **Output**: The vector representation V(t)of the comment text.

$$V(t)=(1/n)*\;\;\;\Sigma \;\;\;h_{i}$$ (2)

Finally, calculate the similarity weight and extract keywords. To measure the similarity between each word and the comment text, this article uses cosine similarity for calculation. Based on the calculated similarity value, we select the top ranked words as the key feature words for this comment statement. Assuming the keyword is w and its vector is represented as *V(w)*, the cosine similarity calculation between the keyword *w* and the comment text *t* is shown in formula (3).

- **Input**: The vector representation V(w) of the keyword and the vector representation V(t) of the comment text.
- **Output**: The similarity weight sim(w,t) between the keyword w and text t.

sim(w,t)=(V(w)·V(t))/(∥V(w)∥*∥V(t)∥) (3)

where, *sim (w, t)* represents the similarity weight between keyword *w* and text *t*.

In summary, KeyBert uses the Bert model to encode text into vector representations and uses cosine similarity to calculate the similarity between keywords and text. By calculating formulas and sorting operations, a list of keywords in the text can be obtained.

### 3.2. Construction of keyword vector based on Word2Vec

The principle of Word2Vec is to use the idea of deep learning to transform the processing of text content into vector operations in high-dimensional vector spaces, and to transform semantic similarity of text into spatial vector similarity. The core idea is to train words into high-dimensional real number vectors. The vector contains rich word information, so synonyms can be found by calculating similarity through the vector distance between words[49]. The basic steps for constructing keyword vectors based on Word2Vec are as follows:

Firstly, the comment text is segmented using natural language processing techniques, and the segmentation results can be used as a corpus.

- **Input**: The raw comment text.
- **Output**: A segmented corpus of words.

Secondly, Word2Vec is used to train keyword vectors and convert keywords in comments into vector form. The two important models in Word2Vec are the CBOW model and the Skip gram model. Among them, the CBOW model predicts the current word under the premise of knowing the context, which is suitable for situations with small datasets. This article is based on the CBOW model to calculate the word vector of keywords, and the calculation process is shown in formula (4).

- **Input:** The keywords extracted using KeyBERT.
- **Output**: The vector representation V(w) for each keyword.

V(w)=f(context) (4)

Where V(w) is the word vector that needs to be predicted, and “context” refers to the surrounding words of (w).

### 3.3. Construction of evaluation index system based on K-means

#### (1) Extraction of satisfaction evaluation indicators for green products.

In order to extract satisfaction evaluation indicators for green products, we used the K-means method to cluster keywords. In our study, *k* represents the number of keyword clusters. The specific steps for extracting consumer satisfaction indicators based on the K-means method are as follows:

1) Using the elbow method to determine the *k* value, it is desired to cluster the feature words to obtain *k* sets.
2) Select the initialized *k* word vectors as the clustering center.
3) For each feature word vector in the datasets, calculate its Euclidean distance from each center to obtain clusters corresponding to *k* centers. On this basis, the first round of clustering is finished.
4) Update the mean vector of the cluster class based on the cluster to which each word vector belongs.
5) Repeat steps (3) and (4). Until a certain termination condition is reached, the clustering of feature words is completed.
6) Select the top eight feature words with similarity scores from various clusters to define the evaluation dimension.

#### (2) Calculation of the weight of satisfaction evaluation indicators for green products.

The *k* evaluation indicators can be obtained through K-means clustering, and the weights of each indicator are determined based on the KeyBert similarity score of the feature words under each indicator after clustering. The specific calculation steps are as follows:

Firstly, according to formula (3), the sum of similarity weights for keyword *w* in all texts can be calculated, and the average similarity weight for keyword *w* can be obtained. The calculation is shown in formula (5):


$$sim(w)=\frac{sim(w,t_{1})+sim(w,t_{2})+...+sim(w,t_{n})}{n}$$ (5)

Among them, $sim\left(w,t_{n}\right)$ represents the similarity weight of keyword *w* in the *n-*th text *t*, and $sim\left(u_{w}\right)$ represents the average similarity weight of keyword *w* in all texts.

Secondly, for each evaluation indicator *u*, calculate the sum of the similarity weights sum_w(u) of all its feature keywords, as shown in formula (6). For the evaluation index system *z*, calculate the sum of all evaluation index weights sum_u(z), as shown in formula (7):


$$sum_{u}(w)=\sum (sim(u_{w_{i}}))=sim(u_{w_{1}})+sim(u_{w_{2}})+...+sim(u_{w_{i}})$$ (6)

$$sum_{z}(u)=\sum (sum_{z}(u_{j}))=sum_{z}(u_{1})+sum_{z}(u_{2})+...+sum_{z}(u_{j})$$ (7)

Where, $sim\left(u_{w_{i}}\right)$ represents the similarity weight of the *w*_*i-*_th keyword in the evaluation index *u*, and $sum\_z\left(u_{j}\right)$ represents the sum of the similarity weights of all keywords in the *j-*th evaluation index $u_{j}$ in the evaluation index system.

Finally, by performing normalization, we can obtain the proportion of each evaluation indicator *u*, which is the weight value *w(u)* of the evaluation indicator *u*, as shown in formula (8):


$$w(u)=\frac{sum_{u}(w)}{sum_{z}(u)}.$$ (8)

**Fig 1:**
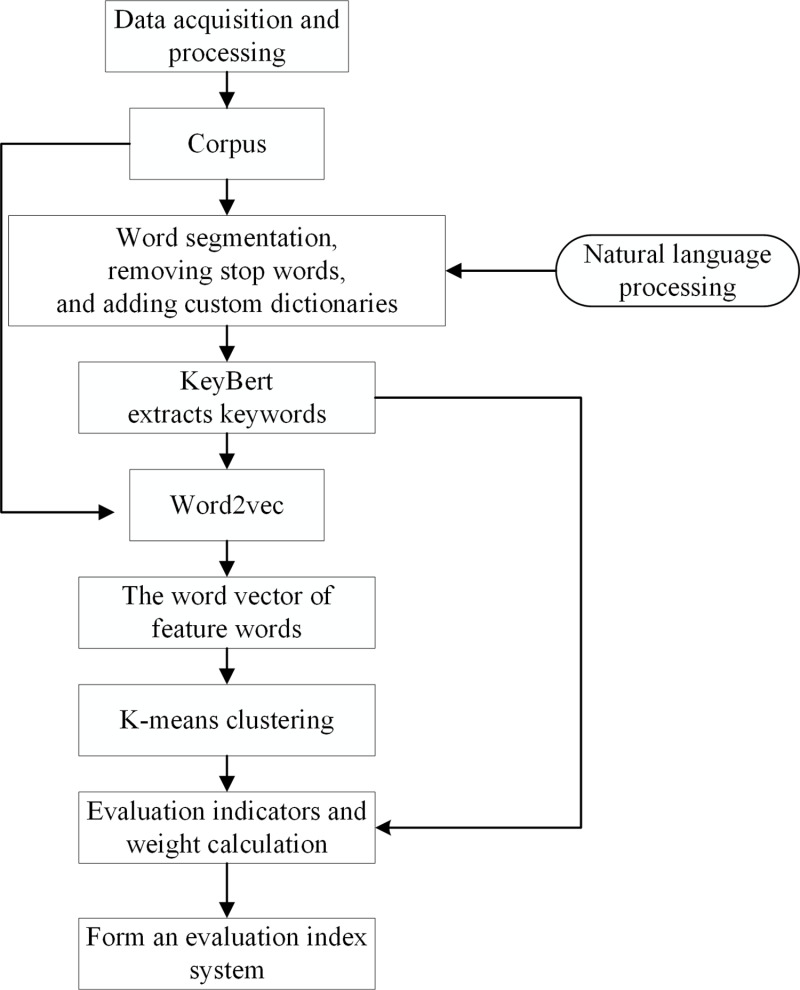
The construction process of consumer satisfaction evaluation index system for green products.

Therefore, the construction process of the evaluation index system for consumer satisfaction of green products based on text mining is shown in Fig 1.Please refer to S1 Table.

## 4. Empirical research

In July 2022, the State Council Executive Meeting called for multiple measures to expand consumption and identified multiple measures to support the consumption of green and intelligent home appliances. Our study selected green and energy-saving household appliances as the empirical research object, and selected online comments on energy-saving air conditioning on the JD platform as empirical data.

### 4.1. Construction of keyword database

We used a web-scraping tool to collect approximately 11,000 user feedback entries on level 1 energy-saving air conditioning.Firstly, perform data preprocessing. We screened text with comment length greater than 15 but less than 200 and preprocessed the data, including data cleaning, word segmentation, and removing stop words. Then, this article used the KeyBert method to filter out the top five nouns, gerunds, verbs, and adjective phrases with higher weights in each comment text as keywords for the evaluation index system. We conducted word frequency analysis on the selected keywords and selected keywords with a frequency greater than 10 to construct a core feature word database. Some high-frequency keywords are shown in Table 1.

**Table 1:** Partial keywords and word frequency.

Keyword	Word frequency	Keyword	Word frequency	Keyword	Word frequency
Installation	1784	Good-looking	645	Speed	405
Outward appearance	1298	Logistics	635	After Sales	351
Quality	1240	Product	615	Fashion	348
Brand	1149	Cost performance	578	Heating	332
Energy saving	1073	Shape	553	Muting	332
Refrigeration	675	Deliver goods	510	Appearance	332
Service	652	Cold and warm	481	Energy efficiency	278

The data collection and analysis methods used in this study adhered to the terms and conditions of the data source to ensure compliance and ethical standards.

We gathered a dataset comprising 16,000 user feedback entries pertaining to Level 1 energy-saving air conditioning units.Initially, we performed data preprocessing, screening texts with comment lengths greater than 15 and less than 200 characters. This preprocessing included data cleaning, word segmentation, and the removal of stop words.In the next step, we employed the KeyBert method to identify the top five keywords from each comment text, specifically focusing on nouns, gerunds, verbs, and adjective phrases that carried higher weights. The consideration of Part-of-Speech (POS) tagging during this process is crucial, as the POS provides insights into the role and function of keywords within sentences. The POS of a keyword directly influences its function; for example, nouns typically serve as subjects or core concepts, while verbs often indicate actions or behaviors. By analyzing the POS of keywords, we can gain a better understanding of their true meaning and role in the sentence, thereby accurately capturing the themes and core content of the text.Following this, we conducted a word frequency analysis on the identified keywords and retained those with a frequency greater than 10 to construct a core feature word database. Some high-frequency keywords are presented in Table 1. This methodology not only refined our keyword selection but also ensured that the keywords accurately reflected consumer sentiments regarding energy-saving air conditioning products.

### 4.2. Word vectorization of feature keywords

Based on the neural network model, our study utilized the CBOW model and mapped words into a distributed vector based on contextual information and the internal structure of sentences. Based on the word2vec model and comment data corpus, we transformed the feature words in the core feature word lexicon into high-dimensional word vectors. Among them, the partial data represented by keyword vectorization is shown in Table 2.

**Table 2:** Partial data representation of word2vec word vectorization.

Number	Keyword	Word vector
1	Air-conditioning	[-0.0630, -0.0855, 0.0476,......, 0.2836, -0.3224]
2	Installation	[-0.1287, 0.1829, -0.0384,......, -0.4320, -0.1212]
3	Quality	[-0.1516, -0.0756, 0.3992,......, 0.0661, -0.1628]
……	……	……
300	Service	[0.1078, -0.1329, -0.0315,......, 0.2660, -0.5206]

### 4.3. Clustering of feature keywords and definition of evaluation indicators

We determined the optimal number of clusters using the SSE elbow method and used the sum of squared errors generated by model iteration to determine the appropriate clustering points. The results showed that the optimal number of clusters was around 7–8. After determining the optimal number of clusters through SSE, we calculated the Euclidean distance from the word vector of the keywords to the nearest cluster center point, and formed clusters for the keywords. Each clustering theme in the clustering results can form a green product consumer satisfaction evaluation index. The final clustering results are shown in Table 3.

**Table 3:** The top eight feature keywords.

Category	Feature keyword	Evaluating indicator
1	Appointment, attitude, communication, responsibility, speed, patience, punctuality, handling	Service
2	Installation, installed, master, proficiency, install equipment, efficiency, punching, speed	Installation
3	Logistics, delivery, express delivery, distribution, receiving, shipping, repair, freight	logistics
4	Energy efficiency, wind power, energy consumption, wind speed, air volume, power, sweeping, temperature	Function
5	Energy saving, frequency conversion, keep warm, cooling, heating, silence, refrigeration, cold air	
6	Appearance, shape, fashion, beauty, aesthetics, color, simplicity, styling	Appearance
7	Service, dedication, thoughtfulness, satisfaction with service, reputation, positive feedback, excellent service, trust	Service
8	Affordable, discounted, cost-effective, cost performance, cheap, free, worth, price	Price

We determined the optimal number of clusters using the Sum of Squared Errors (SSE) elbow method, which quantitatively represents the degree of sample aggregation. A smaller SSE value indicates a tighter grouping of samples within each cluster[50]. The SSE is calculated using the following formula:


$$SSE=\sum_{i=1}^{k}\sum_{n\in C_{i}}|n-m_{i}{|}^{2}$$ (9)

Where *C*_*i*_ represents the *i-th* cluster, *n*_*i*_ denotes the number of sample points in *C*_*i*_,and *m*_*i*_ is the mean of all samples within *C*_*i*_.As the number of clusters 𝑘 increases, the degree of aggregation within each cluster tends to improve, leading to a finer partitioning of the samples and a corresponding reduction in the SSE.

Initially, when 𝑘 is less than the true number of clusters, an increase in 𝑘 rapidly enhances the aggregation within each cluster, resulting in a significant decrease in SSE. However, once 𝑘 reaches the true number of clusters, further increases yield diminishing returns in terms of aggregation, causing the SSE reduction to level off. Consequently, the relationship between SSE and 𝑘 typically forms an elbow shape, with the elbow corresponding to the true number of clusters in the data.

The final clustering results indicated an optimal number of clusters around 7–8. After establishing the optimal cluster count through the SSE method, we calculated the Euclidean distance from the word vectors of the keywords to the nearest cluster centroids, thereby forming clusters for the keywords. Each clustering theme identified in the results contributes to the development of a consumer satisfaction evaluation index for green products. The final clustering results are presented in Table 3.

### 4.4. Weight calculation and analysis of evaluation indicators

Firstly, the KeyBert model was used to calculate the similarity of feature keywords in each category.

Secondly, we calculated the sum of the similarity of keywords in each category and normalized them, as shown in Table 4.

**Table 4:** Similarity weights of feature keywords for each category.

	Category 1		Category 2		Category 3		Category 4		Category 5		Category 6		Category 7		Category 8	
1	satisfaction	292.60	installation	754.25	logistics	304.22	energy efficiency	110.06	energy saving	474.17	appearance	582.97	service	291.16	cost performance	286.04
2	appointment	68.21	master	354.48	delivery	221.40	temperature	32.37	refrigeration	297.53	shape	241.12	positive feedback	200.46	worth	207.41
3	attitude	63.72	speed	175.20	express delivery	67.45	sweeping	18.13	silence	148.25	fashion	167.23	service attitude	181.33	affordable	163.44
4	responsibility	37.74	efficiency	28.56	distribution	60.46	wind power	15.20	heating	143.30	beauty	159.84	trust	94.63	price	80.74
5	arrangement	29.97	already installed	24.75	receiving	36.67	energy consumption	11.75	keep warm	31.14	aesthetics	135.21	excellent service	28.16	cost-effective	53.03
6	patience	17.05	proficiency	12.78	shipping	16.37	wind speed	10.85	frequency conversion	8.55	color	69.81	thoughtfulness	10.21	cheap	49.40
7	communication	13.54	punching	8.75	repair	10.80	air volume	8.76	cooling	9.84	simplicity	16.77	reputation	10.12	discounted	35.16
8	punctuality	11.77	install equipment	7.53	freight	4.87	power	4.84	cold air	7.46	styling	11.74	dedication	5.80	free	23.64
Summation	534.6		1366.31		722.24		211.96		1120.23		1384.7		821.87		898.85	
Normalization	0.076		0.194		0.102		0.03		0.159		0.196		0.116		0.127	

Finally, we calculated the weight of each evaluation indicator based on its category. The final results are as follows: the weight of installation evaluation indicators is 27%, logistics evaluation indicators are 10.2%, functional evaluation indicators are 18.9%, appearance evaluation indicators are 19.6%, service evaluation indicators are 11.6%, and price evaluation indicators are 12.7%.

Among the six evaluation indicators, consumers attach great importance to the installation, appearance, and green functions of energy-saving air conditioners. When consumers consider purchasing energy-efficient air conditioners, their emphasis on these three indicators far exceeds other factors. The functionality of energy-saving air conditioners is mainly reflected in their energy efficiency, refrigeration and heating effects, frequency conversion, and other aspects. It is the core manifestation of their green attributes and directly affects the effectiveness of products in daily use. In terms of the installation of energy-saving air conditioners, consumers hope to be able to install them in a timely manner within the scheduled time after purchasing. At the same time, installation efficiency and the proficiency of installation technicians will have a significant impact on the consumer experience. The appearance of energy-saving air conditioners is also a highly valued aspect by consumers. Consumers not only hope that the product has the functional characteristics, but also hope that energy-

saving air conditioners have a beautiful appearance design that can be coordinated and matched with home decoration. In addition, price, logistics, and service are also factors that consumers need to consider when making purchases. Our empirical research was conducted on the JD e-commerce platform, which can achieve standardization and uniformity in services, logistics, and pricing. Therefore, in these three categories of indicators, consumer attention is relatively low.

## 5. Conclusions

In this study, text mining methods were employed to extract keywords, compute word vectors, and cluster comment data from user reviews on e-commerce platforms, culminating in the construction of an evaluation index system for consumer satisfaction with green products. It was demonstrated that user-generated online comment data contains rich information that more comprehensively and objectively reflects consumption and usage experiences compared to traditional survey methodologies. Through the application of KeyBert, word2vec, and K-means methods, keywords were extracted from the comment texts, revealing that consumer satisfaction with energy-saving air conditioning products is primarily assessed based on factors such as installation, appearance, functionality, logistics, price, and service. Notably, it was observed that consumers placed the greatest emphasis on installation, appearance, and functional characteristics, suggesting that manufacturers should prioritize these aspects in product development.However, certain limitations were acknowledged, including the prevalence of false reviews in online comments, which may compromise the objectivity of the data. Furthermore, the study was restricted to online comment data sourced from JD’s e-commerce platform, indicating that future research should aim to explore a broader range of high-quality comments across various platforms.The proposed method distinguishes itself from prior works by integrating advanced text mining techniques to provide a more nuanced understanding of consumer sentiment, thereby highlighting the critical role of online reviews in shaping product evaluations. Previous studies in the field, have utilized keyword extraction and clustering methods but often rely on basic frequency-based approaches, limiting their ability to capture the deeper relationships between words and their contextual meanings. In contrast, our method leverages word embeddings generated by word2vec to capture semantic relationships between keywords, providing a more comprehensive understanding of consumer sentiment.

The contributions of the author include the development of a robust framework for analyzing consumer feedback, which can be adapted for future research across diverse product categories.

## Supporting information

S1 Table Relevant data underlying the findings described in manuscript. (XLSX)
